# The Mechanism of “Treating Different Diseases with the Same Treatment” by Qiangji Jianpi Decoction in Ankylosing Spondylitis Combined with Inflammatory Bowel Disease: A Comprehensive Analysis of Multiple Methods

**DOI:** 10.1155/2024/9709260

**Published:** 2024-05-21

**Authors:** Xuhong Zhang, Lamei Zhou, Xian Qian

**Affiliations:** ^1^Wuxi Affiliated Hospital of Nanjing University of Chinese Medicine, Wuxi, China; ^2^Affiliated Hospital of Nanjing University of Chinese Medicine, Nanjing, China

## Abstract

**Background:**

Ankylosing spondylitis (AS) and inflammatory bowel disease (IBD) are prevalent autoimmune disorders that often co-occur, posing significant treatment challenges. This investigation adopts a multidisciplinary strategy, integrating bioinformatics, network pharmacology, molecular docking, and Mendelian randomization, to elucidate the relationship between AS and IBD and to investigate the potential mechanisms of traditional Chinese medicine formulations, represented by Qiangji Jianpi (QJJP) decoction, in treating these comorbid conditions.

**Methods:**

We utilized databases to pinpoint common targets among AS, IBD, and QJJP decoction's active compounds through intersection analysis. Through Gene Ontology (GO) and Kyoto Encyclopedia of Genes and Genomes (KEGG) pathway analyses, we mapped a network in Cytoscape, isolating critical targets. Molecular docking with AutoDock validated the affinity between targets and compounds. ROC analysis and dataset validation assessed diagnostic performance, while Gene Set Enrichment Analysis (GSEA) offered pathway insights. Mendelian randomization explored the AS-IBD causal relationship.

**Results:**

Screening identified 105 targets for QJJP decoction, 414 for AS, and 2420 for IBD, with 85 overlapping. These targets predominantly participate in organismal responses and DNA transcription factor binding, with a significant cellular presence in the endoplasmic reticulum and vesicle lumen. Molecular docking, facilitated by Cytoscape, confirmed IL1A, IFNG, TGFB1, and EDN1 as critical targets, with IFNG demonstrating diagnostic potential through GEO dataset validation. The integration of GSEA with network pharmacology highlighted the therapeutic significance of the relaxin, osteoclast differentiation, HIF-1, and AGE-RAGE signaling pathways in QJJP decoction's action. Mendelian randomization analysis indicated a positive causal relationship between IBD and AS, pinpointing rs2193041 as a key SNP influencing IFNG.

**Conclusion:**

Based on the principle of “treating different diseases with the same method” in traditional Chinese medicine theory, we explored the intricate mechanisms through which QJJP decoction addresses AS and IBD comorbidity. Our research spotlighted the pivotal role of the IFNG gene. IFNG emerges not only as a key therapeutic target but also assumes significance as a potential diagnostic biomarker through its genetic underpinnings. This investigation establishes a solid base for subsequent experimental inquiries. Our findings introduce novel approaches for incorporating traditional Chinese medicine into the treatment of AS-IBD comorbidity, setting the stage for groundbreaking research directions.

## 1. Introduction

Ankylosing spondylitis (AS) is a chronic immune systemic disease characterized primarily by inflammation of the axial skeleton, which can also involve peripheral joints [1]. Epidemiological studies have found that the global prevalence of AS ranges from 0.1% to 1.4% and is higher in men than in women [2]. In addition to skeletal lesions, AS can involve other organ lesions, most commonly uveitis, psoriasis, and intestinal inflammation [3], with the latter being the most common complication with a prevalence of up to 40% to 60% [4]. This intestinal inflammation has a 5% to 20% chance of developing into inflammatory bowel disease (IBD) over time [5], and indeed, there are studies demonstrating that the incidence of IBD is not uncommon in AS [6]. Inflammatory bowel disease is also an immune disease that primarily affects the digestive system and includes multiple forms, with ulcerative colitis (UC) and Crohn's disease (CD) predominating [7]. The prevalence of IBD has increased from 0.3% to 1.3% in the last decade, with an upward trend year over year [8]. The clinical symptoms of IBD are mainly rectal bleeding, abdominal pain, diarrhea, and weight loss [9]. In addition, IBD is often associated with various extraintestinal manifestations (EIMs), such as ocular, cutaneous, hepatobiliary, and hematological, all of which can be involved [10]. At the same time, arthropathy is also a common EIM, with an incidence of up to 40% [11] and being more common in CD than UC [12]. Although clinical studies indicate that comorbidity between these two diseases is common, their causal relationship remains unclear. Furthermore, since the diagnosis and treatment of these two diseases are typically managed by separate departments, comorbid patients not only fail to achieve the desired treatment outcomes but also face increased financial burdens and even potential elevated mortality risks. Therefore, exploring the causal relationship between these two diseases and combining the use of traditional herbal medicine interventions to improve clinical symptoms become of paramount importance.

Chinese herbal medicine contains a variety of compounds and targets. Compound preparation is one of the methods of traditional Chinese medicine used to treat complex diseases with improved curative properties and fewer adverse effects [13]. The concept of “luo mai” (collateral vessels) was mentioned in the Huangdi Neijing (Yellow Emperor's Inner Canon) and has been continuously inherited and summarized by various medical practitioners, gradually forming a relatively complete therapeutic system known as “collateral theory.” During the Qing Dynasty, physician Ye Tianshi further proposed theories regarding “chronic diseases entering the collateral vessels” and “chronic pain entering the collateral vessels,” enriching the theoretical basis of this system. In modern times, many medical practitioners have achieved remarkable clinical outcomes by applying the concept of “luo mai” to treat various complex conditions. Building upon the theory of “luo mai” and the principle of “treating different diseases with the same method,” our team has successfully utilized Qiangji Jianpi (QJJP) decoction to treat patients with AS combined with IBD, resulting in favorable clinical results. However, the underlying mechanism of this decoction remains unclear. Therefore, in this study, we will employ various research methods, including network pharmacology, molecular docking, and bioinformatics, to explore the potential mechanism, providing a theoretical foundation for further investigation.

Network pharmacology is one field of pharmacology that uses omics and network visualization technologies to demonstrate the complex biological network relationships between drugs, genes, disorders, and targets, as well as to predict drug pharmacological action mechanisms [14]. The network pharmacology technique has been widely employed in target research in traditional Chinese medicine and has considerable guiding relevance for explaining its pharmacological processes. Molecular docking is a structure-based computer modeling approach. It is commonly used to aid in the development of novel drugs [15]. Thanks to advancements in high-throughput sequencing and microarray technology in recent years, bioinformatics analysis has become a valuable tool for identifying new genes and biomarkers associated with various diseases, including autoimmune diseases [16]. Therefore, based on the combination of the above three methods, this study explores the target effect of QJJP decoction in treating AS comorbid IBD and predicts the possible mechanism of its use.

Mendelian randomization (MR) is an analytical method used to infer potential causal relationships [17]. This approach utilizes single nucleotide polymorphisms (SNPs) as instrumental variables (IVs) to assess the causal links between exposure factors and study outcomes [18], without being adversely affected by reverse causation [19]. The core principles of MR entail the robust association between the selected instrumental variable and the exposure, the absence of associations with confounding factors, and its exclusive impact on the outcome through its effect on the exposure. These principles collectively ensure dependable causal inference. Therefore, we employed this method to investigate whether a causal relationship exists between AS and IBD.

**Figure 1:**
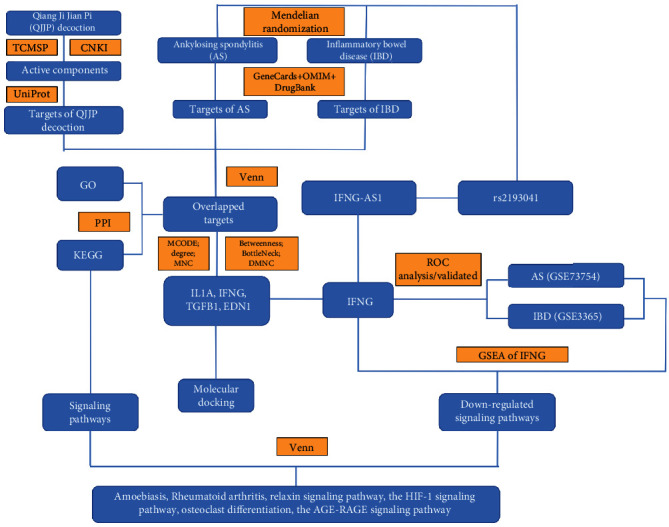
The workflow of this study.

The study workflow is shown in Figure 1.

## 2. Materials and Methods

### 2.1. Data Preparation

The composition of QJJP decoction includes the following herbs: Fang Feng (Radix Saposhnikoviae), Bai Zhi (Radix Angelicae Dahuricae), Wei Ling Xian (Radix Clematidis), Chuan Niu Xi (Radix Achyranthis Bidentatae), Dang Gui (Radix Angelicae Sinensis), Du Huo (Radix Angelicae Pubescentis), Ju He (Exocarpium Citri Grandis), Sang Ji Sheng (Ramulus Taxilli), Bai Zhu (Rhizoma Atractylodis Macrocephalae), Bai Shao (Radix Paeoniae Alba), Di Jin Cao (Herba Lycopodii Clavati), Gan Jiang (Rhizoma Zingiberis), and Gan Cao (Radix Glycyrrhizae). The Traditional Chinese Medicine Systems Pharmacology (TCMSP, https://old.tcmsp-e.com/tcmsp.php/) [20] database is a pharmacology database which provides information about active herb components and their corresponding targets. On this platform, we searched for the chemical components of “baishao”, “baizhu”, “baizhi”, “chuanniuxi”, “danggui”, “dijingcao”, “duhuo”, “fangfeng”, “gancao”, “juhe”, “sangjisheng”, “weilingxian”, and “ganjiang”, using these Chinese Pinyin names as keywords. The collected chemical components were screened according to the two following conditions: oral bioavailability (OB) ≥ 30% [21] and drug‐likeness (DL) ≥ 0.18 [22]; the relevant literature was searched to supplement the target results through the China National Knowledge Infrastructure (CNKI) platform using “juhe” as the keyword. After screening, the UniProt database (https://www.uniprot.org/) [23] was used to standardise the protein target information of the compounds.

Details on human targets associated with AS and IBD were collected from the following sources: GeneCards (http://genecards.org) [24], Online Mendelian Inheritance in Man (http://www.omim.org) [25], and DrugBank databases (http://www.drugbank.ca) [26]. “Ankylosing Spondylitis” and “Inflammatory Bowel Disease” were used as the keywords to collect known targets from these databases for the species *Homo sapiens*. The disease targets retrieved from different databases were integrated, and duplicate records were removed to obtain candidate AS and IBD targets.

### 2.2. Construction of Overlapping Target-Active Compound Network

The website (http://www.bioinformatics.com.cn/) was utilized to identify the overlapping targets among QJJP decoction, ankylosing spondylitis (AS), and inflammatory bowel disease (IBD). The overlapping targets and their corresponding active compounds were uploaded into the Cytoscape [27] to construct and visualize the “overlapping target-active compound” network and visualize it. In the network, nodes represented compounds and targets, while edges indicated the interactions between them.

### 2.3. GO and KEGG Enrichment Analyses

To further identify the potential mechanisms and functions, we performed Gene Ontology (GO) term analysis [28] and Kyoto Encyclopedia of Genes and Genomes (KEGG) pathway [29] enrichment analysis of the overlapped targets carried out by using the “clusterProfiler” [30] in R (v. 4.2.2). The results were visually presented, and a significance threshold of *P* values < 0.05 was used to determine statistical significance.

### 2.4. PPI Network and Hub Target Analysis

The screened overlapped targets were uploaded onto the STRING online tool (https://string-db.org/) [31], a data platform that hosts almost all known and predicted interactions between different proteins. The species parameter was set to *Homo sapiens*, while the confidence score was set at 0.9. Subsequently, a protein-protein interaction (PPI) network was constructed by hiding disconnected nodes. The network was visualized and analyzed using Cytoscape. Key modules were identified using the Molecular Complex Detection (MCODE) app, with a score threshold set to ≥10. Additionally, other significant topological parameters including DMNC (density of maximum neighborhood component), MNC (maximum neighborhood component), degree, BottleNeck, and betweenness were calculated using the CytoHubba App in Cytoscape. Finally, the obtained results were further analyzed, and the hub targets were identified.

### 2.5. Pathway Analysis of Hub Targets

We filtered the significantly enriched pathways based on the hub targets, constructed a network connecting the hub targets with the enriched pathways, and visualized the results.

### 2.6. Hub Target-miRNA Prediction

We utilized the starBase database (http://starbase.sysu.edu.cn/) [32] to estimate the miRNAs of hub targets. The result was visualized via Cytoscape.

### 2.7. Molecular Docking

The binding capability of key compounds with their targets was assessed using molecular docking. Firstly, the three-dimensional (3D) structures of the key compounds were obtained from the Protein Data Bank (PDB) databases (http://www.rcsb.org/) [33]. Next, AutoDockTools software [34] was employed to convert the file formats of the key compounds and their related targets into “pdbqt” format and define the coordinates for their “grid box.” Finally, molecular docking between the key compounds and their targets was performed using AutoDockTools and PyMOL software. A ligand-receptor binding energy below 0 kcal/mol indicates spontaneous binding of the ligand to the protein receptor, while a binding energy below -5.0 kcal/mol suggests a strong binding affinity [35].

### 2.8. Prediction and Validation of Hub Genes

ROC analysis was performed to evaluate the diagnostic performance. AUC values were calculated using the GSE73754 and GSE3365 datasets to distinguish AS and IBD from control samples. AUC values above 0.5 were considered diagnostic, while values above 0.7 indicated good specificity and sensitivity. Additionally, the expression levels of hub genes in AS and IBD patients were compared to normal controls using validated datasets, and the results were visualized using box plots.

### 2.9. GSEA

GSEA (Gene Set Enrichment Analysis) is a method used to analyze the enrichment of specific gene sets. This approach utilizes microarray data obtained from genome-wide expression profiling [36]. Heatmaps were constructed using the GSE73754 and GSE3368 datasets to visualize the differentially expressed genes (DEGs) between the high and low expression groups of IFNG in AS and IBD. Subsequently, GSEA was performed using the “MSigDB” package based on the expression levels of IFNG. Gene sets were considered significantly enriched if the FDR < 0.20 and *P* value < 0.05.

### 2.10. Identify the Hub Signaling Pathway

Based on the findings mentioned above, we visualized the intersection of crucial signaling pathways obtained from network pharmacology and the downregulated signaling pathways identified through GSEA.

### 2.11. Mendelian Randomization

We conducted bidirectional Mendelian randomization (MR) analysis using publicly available genome-wide association study (GWAS) data for ankylosing spondylitis (AS) and inflammatory bowel disease (IBD). SNPs were selected based on genome-wide significance (*P* < 5*E* − 08), independence (*r*^2^ < 0.01), and absence of linkage disequilibrium (LD) in summary statistics. The analysis, performed in R using the “TwoSampleMR” package, employed various methods, including inverse variance weighted (IVW), MR-Egger, weighted median, weighted model, and simple model approaches, to determine the causal relationship between the two conditions.

## 3. Result

### 3.1. Identification of Active Compounds in QJJP Decoction

**Figure 2:**
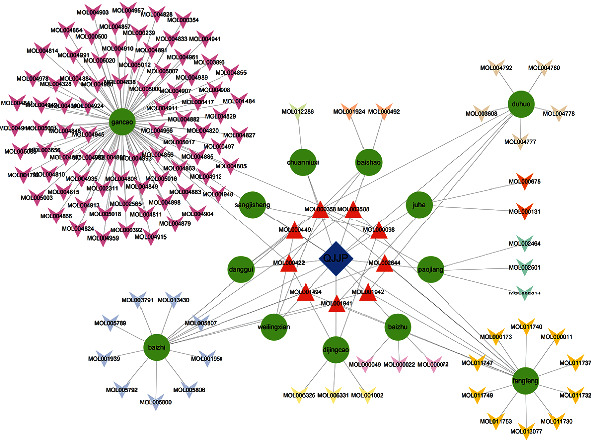
Preliminary screening of QJJP decoction identified active compounds across its components: baishao (13), baizhu (7), baizhi (22), chuanniuxi (4), danggui (2), dijingcao (13), duhuo (9), fangfeng (18), juhe (2), ganjiang (5), sangjisheng (2), weilingxian (7), and gancao (92), with 9 compounds recurrent across these ingredients.

Through the preliminary screening of QJJP decoction, we obtained the following results: there were 13 active compounds in baishao, 7 active compounds in baizhu, 22 active compounds in baizhi, 4 active compounds in chuanniuxi, 2 active compounds in danggui, 13 active compounds in dijingcao, 9 active compounds in duhuo, 18 active compounds in fangfeng, 2 active compounds in juhe, 5 active compounds in ganjiang, 2 active compounds in sangjisheng, 7 active compounds in weilingxian, and 92 active compounds in gancao. Further analysis revealed that a total of 9 compounds were found to be recurrent. The details regarding the Mol ID and molecule name related to the herbs are shown in the supplementary Table S1, as shown in Figure 2.

### 3.2. Obtain Overlapped Targets

**Figure 3:**
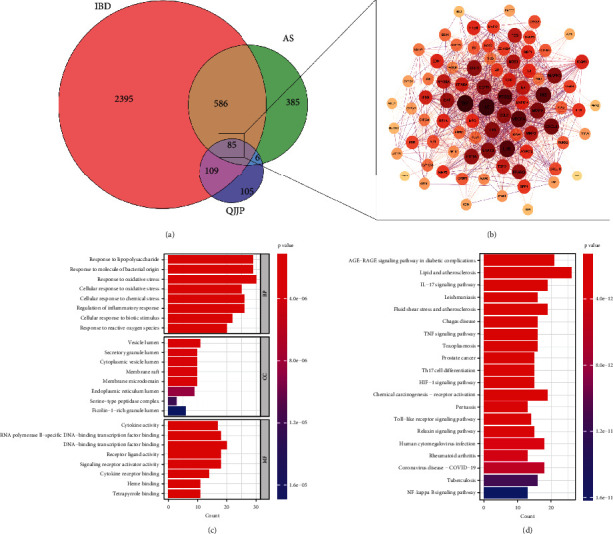
(a) Venny-identified overlapping targets. (b) Network analysis of protein-protein interactions. The larger the node, the darker the colour and the higher the degree. (c) The bar plot shows the overlapping targets enriched in the top 8 terms of BP, MF, and CC ranked by counts. (d) The bar plot shows the overlapping targets enriched in the top 20 KEGCG enrichment pathways ranked by counts.

At first, the drug's active compound and targets were integrated, and duplicates were removed. The following results were obtained: 85 targets in baishao, 18 targets in baizhu, 56 targets in baizhi, 45 targets in chuanniuxi, 50 targets in danggui, 162 targets in dijingcao, 49 targets in duhuo, 74 targets in fangfeng, 50 targets in juhe, 48 targets in ganjiang, 147 targets in sangjisheng, 67 targets in weilingxian, and 221 targets in gancao. We merged these results a second time, deleting duplicate targets, and the total number of targets in the QJJP decoction was 105. Then, for the disease category, the results were integrated and deduplicated by downloading the relevant targets from different websites. The results showed 414 targets in AS and 2420 targets in IBD. Finally, the results of the intersection analysis revealed that 85 targets were overlapped, as shown in Figure 3(a).

### 3.3. PPI Network and Enrichment Analysis

We uploaded these overlapped targets onto the STRING online tool to construct a PPI network, visualized and analyzed by Cytoscape. The result is shown in Figure 3(b). Gene Ontology (GO) and Kyoto Encyclopedia of Genes and Genomes (KEGG) pathway enrichment analyses were performed on 85 overlapped targets. The results showed that 2742 GO terms were significantly enriched, with biological processes (BP), cellular components (CC), and molecular functions (MF) enriched to 2584, 32, and 126, respectively. The top 8 terms of BP, MF, and CC are illustrated in Figure 3(c). BP terms were mainly involved in response to oxidative stress and lipopolysaccharide, response to molecules of bacterial origin, cellular response to oxidative stress, cellular response to chemical stress, and cellular response to the biotic stimulus. CC terms were mainly enriched in vesicle lumen, secretory granule lumen, cytoplasmic vesicle lumen, membrane raft, and membrane microdomain. MF terms were primarily involved in DNA-binding transcription factor binding, RNA polymerase II-specific DNA-binding transcription factor binding, cytokine activity, receptor-ligand activity, and signaling receptor activator activity. In addition, KEGG pathway analysis revealed the enrichment of 170 pathways. These pathways were mainly enriched in lipid and atherosclerosis, IL-17 signaling pathway, TNF signaling pathway, chemical carcinogenesis-receptor activation, AGE-RAGE signaling pathway in diabetic complications, and others. The top 20 signaling pathways are shown in Figure 3(d).

### 3.4. Construction of Herb-Compound-Overlapped Target Network

**Figure 4:**
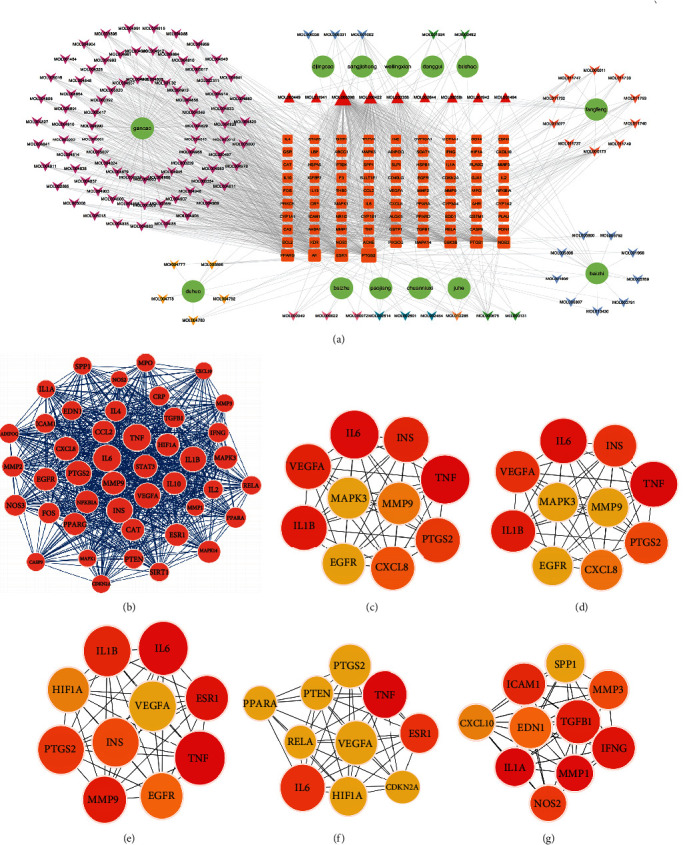
Construction of herb-compound-overlapped target network and hub target analysis: (a) herb-compound-overlapped target network: 226 nodes (128 component nodes, 85 target nodes, and 13 drug nodes) and 1216 edges. The more edges connected to the target, the bigger the shape; (b) MCODE algorithm analysis; (c) degree algorithm analysis; (d) MNC algorithm analysis; (e) betweenness algorithm analysis; (f) BottleNeck algorithm analysis; (g) DMNC algorithm analysis.

The network of herb-compound-overlapped targets was created to comprehensively reveal the molecular mechanism of action of the QJJP decoction in treating AS comorbid IBD. Overlapped targets and their corresponding components and herbs were uploaded into Cytoscape to obtain a network of 226 nodes (128 component nodes, 85 target nodes, and 13 drug nodes) and 1216 edges. As shown in Figure 4(a), the more edges connected to the target, the bigger the shape.

### 3.5. Hub Target Analysis

The MCODE plug-in was used to identify significant target cluster modules, and the most critical module was obtained, which is shown in Figure 4(b). This module contained the top 10 potential hub targets: EDN1, IL1A, TGFB1, MMP2, PPARG, PTEN, IL4, IFNG, CAT, and CRP. The CytoHubba plug-in is also one of the methods for identifying hub targets. Based on the degree algorithm, the top 10 potential hub targets were identified: TNF, IL6, IL1B, VEGFA, INS, PTGS2, CXCL8, MMP9, EGFR, and MAPK3, as shown in Figure 4(c). Based on the MNC algorithm, the top 10 potential hub targets were identified, TNF, IL6, IL1B, VEGFA, INS, PTGS2, CXCL8, MMP9, EGFR, and MAPK3, as shown in Figure 4(d). Based on the betweenness algorithm, the top 10 potential hub targets were identified: TNF, IL6, ESR1, MMP9, IL1B, PTGS2, INS, EGFR, HIF1A, and VEGFA, as shown in Figure 4(e). Based on the BottleNeck algorithm, the top 10 potential hub targets were identified: TNF, ESR1, IL6, PPARA, IF1A, VEGFA, RELA, PTEN, CDKN2A, and PTGS2, as shown in Figure 4(f). Based on the DMNC algorithm, the top 10 potential hub targets were identified: IL1A, MMP1, IFNG, TGFB1, ICAM1, NOS2, MMP3, EDN1, CXCL10, and SPP1, as shown in Figure 4(g).

The results were comprehensively analyzed, and 4 potential hub targets were identified: IL1A, IFNG, TGFB1, and EDN1.

### 3.6. Construction of the Hub Target-Pathway Network

**Figure 5:**
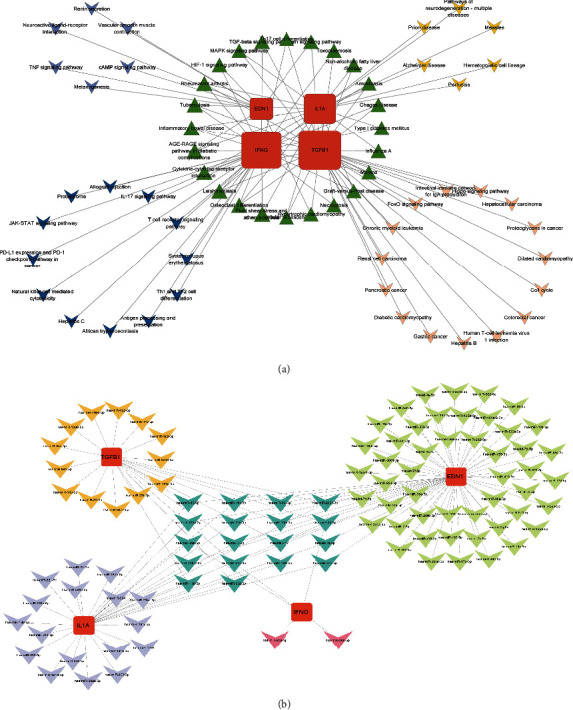
(a) Hub target-pathway network (67 nodes and 95 edges). (b) Hub target-miRNA network (103 nodes and 121 edges). The larger the shapes, the more edges the target connected.

A hub target-pathway network diagram was created to reveal further the role of these 4 hub targets and pathways, as shown in Figure 5(a). Based on this network, 67 nodes and 95 edges were connected; the more edges connected to the target, the bigger the shape. According to the findings, there are 24 recurring pathways, including the MAPK signaling pathway, cytokine-cytokine receptor interaction, Th17 cell differentiation, TGF-signaling pathway, and HIF-1 signaling pathway. These pathways have a high probability of functioning.

### 3.7. Construction of the Hub Target-miRNA Network

We searched the hub targets further to explore the correlation between the hub targets and miRNAs. Through screening, we obtained the following results: 62 miRNAs were obtained for EDN1, 31 miRNAs were obtained for IL1A, 5 miRNAs were obtained for IFNG, and 23 miRNAs were obtained for TGFB1. 17 of these miRNAs were obtained in duplicate, including hsa-miR-210-3p, hsa-miR-27a-3p, hsa-miR-27a-5p, hsa-miR-133a-3p, hsa-miR-24-3p, hsa-miR-532-3p, and hsa-miR-138-5p. The results were uploaded to Cytoscape for visualization, as shown in Figure 5(b).

### 3.8. Molecular Docking

**Figure 6:**
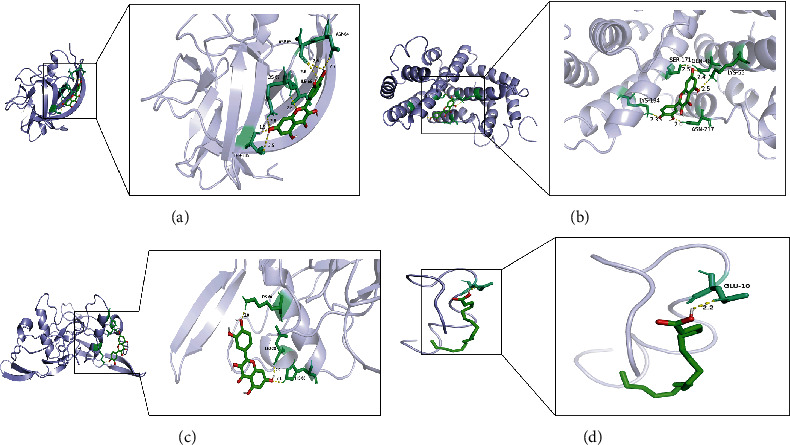
Molecular docking: (a) result of quercetin and ILIA; (b) result of quercetin and IFNG; (c) result of quercetin and TGFBI; (d) result of oleic acid and EDN1.

To validate the results from the above analysis, we performed molecular docking between the 2 key compounds, quercetin (MOL000098) and oleic acid (MOL000675), and their corresponding hub targets. The results showed that the binding energies of 3 pairs (quercetin and IL1A, quercetin and IFNG, and quercetin and TGFB1) were less than -5.0 kcal/mol, indicating that they could bind tightly, as shown in Figure 6. More information about the molecular docking results, including the specific names of the active ingredients and their interactions with the hub targets, is provided in Table 1. Collectively, the molecular docking results suggest that a combination of these targets might play an essential role in the treatment of AS comorbid IBD.

### 3.9. ROC Analysis

**Figure 7:**
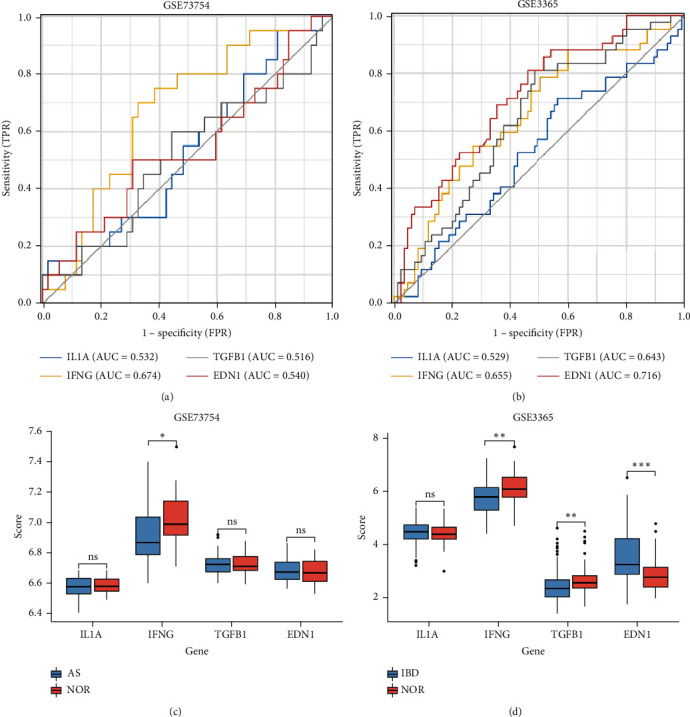
ROC analysis and validated the hub genes: (a, b) AUC values in GSE73754 and GSE3365; (c, d) validation results of 4 hub genes in GSE73754 and GSE3365 datasets (^∗^*P* < 0.05, ^∗∗^*P* < 0.01, and ^∗∗^*P* < 0.001).

ROC analyses were used to validate the diagnostic effectiveness of the biomarkers for AS (GSE73754) and IBD (GSE3365). The AUC values for IL1A, IFNG, TGFB1, and EDN1 in GSE73754 were 0.532, 0.674, 0.516, and 0.540, as shown in Figure 7(a). However, the AUC values for IL1A, IFNG, TGFB1, and EDN1 in GSE3365 were 0.529, 0.655, 0.643, and 0.716, as shown in Figure 7(b). As a result, these 4 hub genes were all diagnostic, but their specificity and sensitivity may have been lower.

### 3.10. Validated the Hub Genes

Then, 4 hub genes were used for expression validation using the GSE73754 datasets (AS) and GSE3365 datasets (IBD). In AS, compared to the control group, the expression level of IFNG in the disease group was downregulated, and there was no statistical difference for IL1A, TGFB1, or EDN1. However, in IBD, IFNG and TGFB1 expressions in the disease group were downregulated relative to the control group, whereas EDN1 expression was elevated, and there was no statistical difference for IL1A, as shown in Figures 7(c) and 7(d). The results showed that IFNG declined in both diseases. So IFNG might be an essential gene.

### 3.11. GSEA of IFNG

**Figure 8:**
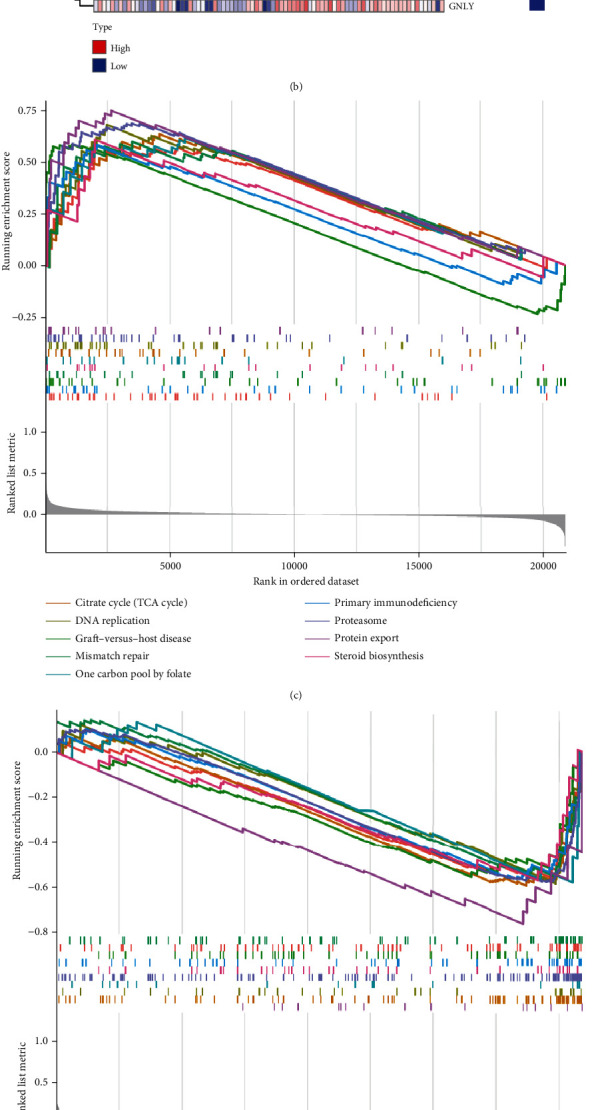
Gene Set Enrichment Analysis: (a, b) heatmaps depict the 30 DEGs between the IFNG high and low groups in AS and IBD. Red indicates that the expression of genes is upregulated, and blue indicates that the expression of genes downregulated; (c) the signaling pathways enrich in the IFNG high expression groups in AS; (d) the signaling pathways enrich in IFNG low expression groups in AS; (e) the signaling pathways enrich in IFNG high expression groups in IBD; (f) the signaling pathways enrich in IFNG low expression groups in IBD.

We chose IFNG in the Gene Set Enrichment Analysis (GSEA) for further study based on the above analysis. Firstly, based on the GSE73754 and GSE3368 datasets, we built two heatmaps to illustrate the differentially expressed genes (DEGs) between the IFNG high and low groups in AS and IBD. The top 30 genes with significant differences between the IFNG high and low expression groups are shown in Figures 8(a) and 8(b). Then, we did the GSEA with IFNG to identify signaling pathways enriched in AS and IBD. In AS disease, a total of 107 signaling pathways were enriched, and 41 were positively regulated, such as steroid biosynthesis, proteasome, DNA replication, citrate cycle (TCA cycle), and graft-versus-host disease; 66 were negatively regulated, such as primary bile acid biosynthesis, B cell receptor signaling pathway, osteoclast differentiation, collecting duct acid secretion, and endometrial cancer. These results are shown in Figures 8(c) and 8(d). In IBD disease, 95 signaling pathways were enriched, and 30 were positively regulated, such as butanoate metabolism, DNA replication, graft-versus-host disease, mannose type O-glycan biosynthesis, and nonhomologous end joining; 65 were negatively regulated, such as linoleic acid metabolism, neutrophil extracellular trap formation, nitrogen metabolism, synaptic vesicle cycle, and systemic lupus erythematosus. These results are shown in Figures 8(e) and 8(f). The GSEA results were complementary to previous studies.

### 3.12. Identify the Hub Signaling Pathway

**Figure 9:**
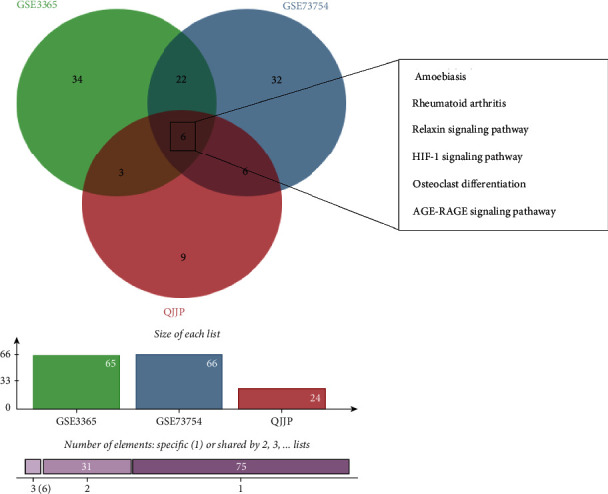
Venn diagram showing the intersection of hub signaling pathway.

We obtained 24 critical signaling pathways from network pharmacology, 66 downregulated critical signaling pathways in AS, and 65 downregulated critical signaling pathways in IBD obtained from GSEA. Then, we intersected these critical signaling pathways. As shown in Figure 9, there were 6 overlapping signaling pathways: amoebiasis, rheumatoid arthritis, relaxin signaling pathway, HIF-1 signaling pathway, osteoclast differentiation, and AGE-RAGE signaling pathway.

### 3.13. Bidirectional Causal Relationship between AS and IBD

**Figure 10:**
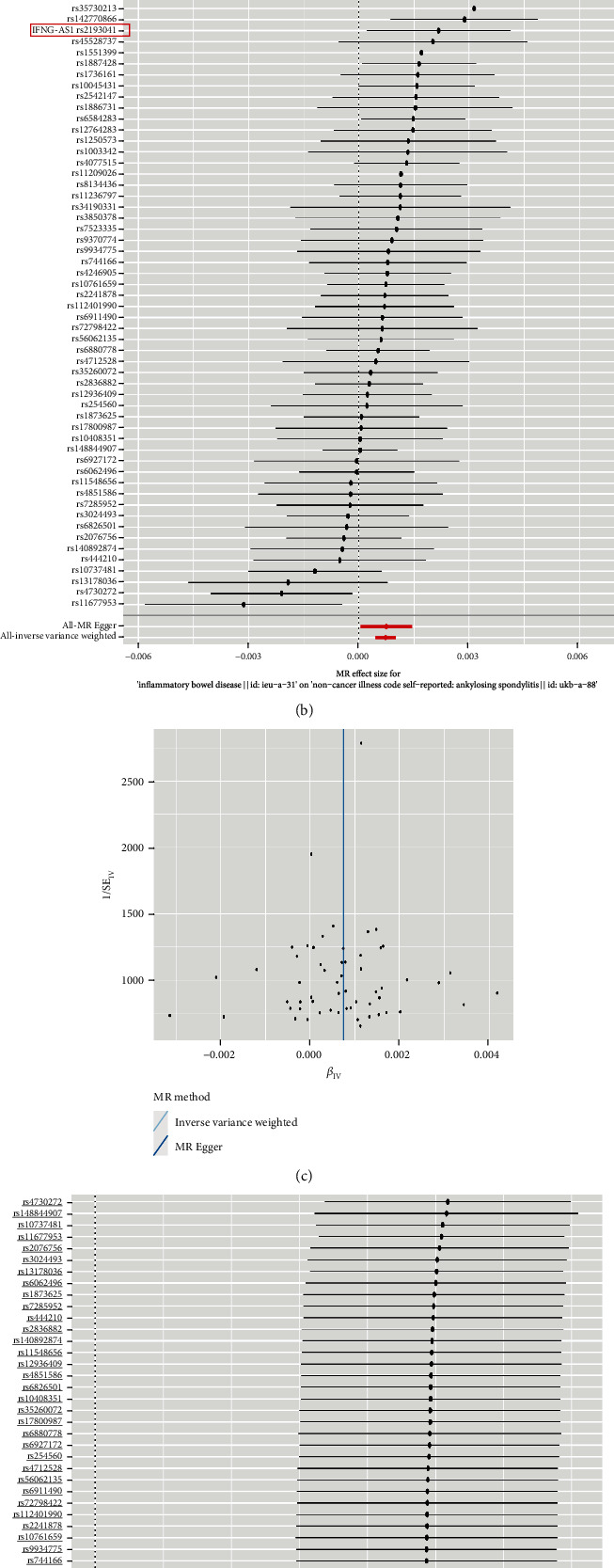
Mendelian randomization study results: (a) scatter plot showing the causal effect of IBD on the risk of AS; (b) forest plot displaying the causal effect of each SNP on AS risk; (c) funnel plot illustrating the overall heterogeneity in MR assessment of the impact of IBD on AS; (d) leave-one-out plot of the causal effect of IBD on AS risk when one SNP is omitted.

In this study, we initially conducted an analysis of the causal relationship between IBD and AS. We extracted 57 SNPs associated with IBD as instrumental variables (IVs), and all these SNPs had *F*-values greater than 10. The study results showed no evidence of weak instrument bias, as illustrated in Figures 10(a) and 10(b). Furthermore, the funnel plot displayed a symmetrical distribution of SNPs, providing further confirmation of the absence of bias in the study, as shown in Figure 10(c). The MR analysis results presented in Table 2 indicated a positive causal association between IBD and the onset of AS. MR-Egger regression analysis yielded a *P* value of 0.95, indicating no presence of horizontal pleiotropy. Finally, validation was performed using a “leave-one-out” approach where individual SNP data points were systematically removed to observe their impact on overall results; however, no strongly influential SNP loci were identified, as illustrated in Figure 10(d). This signifies the validity of the MR findings. Subsequently, we reversed the exposure and outcome functions to validate the causal relationship between AS and IBD using a similar methodology employed in our previous analysis. The MR results are presented in Table 3 for reference purposes. The results showed no statistical significance. Therefore, we conclude that AS does not have a causal effect on the occurrence of IBD.

## 4. Discussion

It is worth noting that if there are comorbidities, such as rheumatoid arthritis with Sjogren's syndrome or ankylosing spondylitis with osteoporosis, the treatment of these comorbidities is relatively mature, and the disease outcome is good [37, 38]. However, interdisciplinary studies on comorbidities are relatively rare, and the prognosis for patients is poor. Both ankylosing spondylitis and inflammatory bowel disease are systemic, progressive, and recurrent chronic diseases [39, 40]. According to epidemiology, the incidence of both diseases is increasing. No matter what type of disease it is, it will seriously affect patients' quality of life, not to mention patients with comorbidities. There is currently no complete cure for AS or IBD, and the mechanism of the link between them is unclear.

In traditional Chinese medicine, there is a principle called “bian zheng shi zhi” which emphasizes the importance of individualized diagnosis and treatment. Within this principle, the application of the same treatment methods for different diseases is known as “yi bing tong zhi” or “treating different diseases with the same method.” It is a flexible application of the “bian zheng shi zhi” principle and serves as a premise for the use of herbal medicine formulations in treating comorbidities. Herbal medicine formulations have shown great efficacy in alleviating clinical symptoms and improving the quality of life for patients with comorbid conditions. There is substantial scholarly evidence that Chinese medicine is effective and has few side effects in the treatment of ankylosing spondylitis or inflammatory bowel disease [41, 42]. Such research is not only limited to single herbs, but there are also a large number of compound preparations [43, 44]. Despite the efficacy of QJJP decoction, its underlying mechanism of action remains unclear. Therefore, our research team conducted this study to provide a preliminary exploration of the mechanisms underlying the co-occurrence of these two diseases. Additionally, we conducted theoretical research on the mechanisms through which QJJP decoction may treat such comorbidities.

It was found that there were 13 herbs and 128 active ingredients in the QJJP decoction. Nine active ingredients were duplicated, including MOL000358 (beta-sitosterol), MOL000422 (kaempferol), MOL000449 (stigmasterol), MOL001494 (mandenol), MOL001941 (ammidin), MOL001942 (isoimperatorin), MOL002644 (phellopterin), MOL003588 (prangenidin), and MOL000098 (quercetin). Combined with the literature review, we found some important active components, such as mandenol may be one of the active ingredients of Yixinyin in treating myocardial infarction [45], beta-sitosterol regulates vascular smooth muscle cell migration through the PPARG/AMPK/mTOR pathway [46], and isoimperatorin could play an important role in antitumor metastasis and inhibiting inflammation [47, 48]. However, at present, there are more studies on quercetin, and a recent review has suggested that quercetin is vital in anti-SARS-CoV-2, antioxidant, anticancer, antiaging, antiviral, and anti-inflammatory activities [49]. So we focused on these compounds in our subsequent studies.

After obtaining overlapped targets, we performed GO and KEGG analyses. The results of the GO enrichment analysis showed that biological processes were mainly related to the relevance of organismal responses, such as the response to oxidative stress, lipopolysaccharides, and reactive oxygen species. A recent in vitro study on ankylosing spondylitis found that KLF4 downregulates FGF21 via the SIRT1/NF-*κ*B/p53 signaling pathway to activate LPS-induced inflammatory damage and oxidative stress in ATDC5 cells [50]. There was also evidence that excessive inflammatory responses and oxidative stress are thought to be the main features of inflammatory bowel disease [51]. This suggests that inflammatory responses and oxidative stress play an important role in comorbidity development. The enrichment sites are mainly located in the endoplasmic reticulum and vesicle lumen, and their function correlates with the binding of the molecule to DNA transfer factors. Therefore, we suspect that exosomes are involved in the biological process, and we hypothesized that patients with AS comorbid IBD could be treated by intervening in the exosomes. The results of an animal experiment showed that miR-21-Exos could be utilized to treat spine osteoporosis in AS [52]. In IBD disease, exosomes can regulate mucosal barrier function and the immune ecology [53]. A meta-analysis showed that circulating miRNAs were dysregulated in AS patients [54]. In addition, one study showed that proinflammatory cytokines elevated miR-10b, which may function as a feedback loop to inhibit IL-17A by targeting MAP3K7. miR-10b was a promising treatment target in AS patients for inhibiting pathogenic Th17 cell activity [55]. Similarly, several miRNA functions have been discovered in IBD; one in vitro and one in vivo experiment suggested that the miR155/HBP1 axis activates the Wnt/*β*-catenin signaling pathway, causing intestinal fibrosis [56]. Last but not least, as a promising treatment, a treatment technique for exosomes and exosome-like nanoparticles using extracellular vesicles as the carrier was rapidly developed, and good feedback effects were obtained [57].

Subsequently, we have obtained 4 critical targets, IL1A, IFNG, TGFB1, and EDN1, through the combination of overlapped target reconfiguration of drug components and computational methods in Cytoscape. Molecular docking revealed that the key active compounds had a high affinity for these 4 hub targets, implying that QJJP decoction could be treated by targeting IL1A, IFNG, TGFB1, and EDN1. This also validated the reliability of network pharmacology in this study.

Immediately afterwards, we utilized bioinformatics techniques to validate the hub targets and ultimately identified an extremely critical target gene: IFNG. This gene encodes a type II interferon-class soluble cytokine. Both cells of the innate and adaptive immune systems release the encoded protein. The active protein attaches to the interferon gamma receptor as a homodimer, triggering a cellular response to viral and microbial infections. Mutations in this gene are linked to increased susceptibility to bacterial, viral, and parasite infections, as well as several autoimmune illnesses. Subsequently, IFNG was used for GSEA. The downregulation of IFNG was the main pathway associated with these two diseases. By intersecting these pathways with the important pathways of network pharmacology, 6 pathways were obtained: amoebiasis, rheumatoid arthritis, relaxin signaling pathway, osteoclast differentiation, HIF-1 signaling pathway, and AGE-RAGE signaling pathway.

Relaxin can inhibit profibrotic cytokines and/or growth factors but not normal or unstimulated fibroblast proliferation, differentiation, or matrix production. Furthermore, relaxin could augment matrix degradation through its ability to upregulate the release and activation of various matrix-degrading matrix metalloproteinases and/or be able to downregulate tissue inhibitors of metalloproteinase activity [58]. Relaxin mediates many biological actions, including antifibrotic, anti-inflammatory, antiapoptotic, vasodilatory, angiogenic, and organ-protective effects across a range of tissues [59]. More recently, another study has shown that relaxin is considered a potential anti-inflammatory treatment to counteract gut damage in humans affected by inflammatory bowel disease [60]. Osteoclast differentiation is a key event leading to joint destruction in common pathological bone disorders. Some scholars have found that IL-17 induces RANKL expression by osteoblasts, which further promotes osteoclast maturation [61]. In IBD, intestinal inflammation led to altered osteoclast precursor expression of surface receptors involved in osteoclast differentiation and function [62]. As a result, we suspect that QJJP decoction may act as an anti-osteofibrosis agent and reduce intestinal inflammation via the relaxin signaling pathway.

Advanced glycation end products (AGEs) are mostly produced during chronic hyperglycemia or aging. AGEs interact with their receptor RAGE, activating a variety of signal transduction pathway genes and proteins. The AGE-RAGE signaling pathway is associated with various pathological conditions, including diabetes, cardiovascular diseases, and cancer [63]. According to a bioinformatics study, the TGF-*β* signaling pathway, the Hippo signaling pathway, and the AGE-RAGE signaling pathway are the most relevant pathways for AS progression [64]. A network pharmacological study of Ge Gen Qin Lian (GGQL) in the treatment of ulcerative colitis (UC) discovered that the AGE-RAGE signaling pathway is one of the potentially important enrichment pathways [65].

HIF-1 is composed of HIF-1*α* and HIF-1*β* subunits. It promotes target gene transcription under hypoxia and plays essential roles in cell development, physiological adaptations, and pathological processes. IBD can cause inflammatory hypoxia by causing intestinal inflammation and vascular damage. In this environment, HIF-1*α* acts as a transcription factor that modulates cellular adaptation to low oxygen levels and synergistically functions as an intestinal barrier. HIF-1*α* exerts its function by entering the nucleus, dimerizing with HIF-1*β*, and binding to HIF-1 target genes [66]. Elevated inflammatory factors could activate immune cells residing in the synovium and drive spondyloarthropathies [67]. However, the correlation between AS and a hypoxic environment has not been reported. It has been observed by another research team that low HIF-1*α* expression is associated with elevated IFNG expression in tumor-infiltrating NK cells in humans [68]. IFNG also can exert its effects through the HIF-1 signaling pathway, thereby mitigating the inhibition of hypoxia-inducible factor (HIF) activity in intestinal epithelial cells [69]. Therefore, we hypothesized that dysregulation of IFNG contributes to the occurrence of AS comorbid IBD in the hypoxic environment. So, this will be one of the directions our team will investigate in depth.

Prior clinical observational studies have revealed a coexistence phenomenon between AS and IBD, suggesting a potential overlap of susceptibility loci between the two diseases [70, 71], albeit with no genetic evidence to date. Consequently, elucidating the causal relationship between AS and IBD holds significant importance for the treatment of comorbidities. In this study, we conducted a preliminary exploration of the causal relationship between AS and IBD. Our findings indicate a positive correlation between the occurrence of IBD and the development of AS. However, a clear causal relationship between the progression of AS leading to IBD has not been established, which aligns with the research findings reported by Cui et al. [72]. Based on our MR analysis, we consider rs2193041 to be a significant SNP. It is located near the IFNG-AS1 gene on chromosome 12. IFNG-AS1 is an antisense RNA associated with interferon gamma (IFNG) in the immune system and plays a role in regulating IFNG gene expression levels, which indirectly supports the value of IFNG. Brandtzaeg was the first to propose a potential link between intestinal and joint inflammation, arguing that the homing of intestinal mucosal immune cells to synovial inflammatory sites is a crucial aspect supporting the “gut-joint axis” theory [73]. Intestinal inflammation may increase intestinal permeability, allowing antigens within the intestine to migrate outside, thereby triggering a low-grade systemic inflammatory response. This could potentially impact the progression of arthritic conditions [74]. Additionally, the microbial flora in the intestine can influence the normal function of the immune system [75]. A disturbance in the balance of intestinal microbial flora can lead to abnormal immune responses, thereby increasing the risk of AS occurrence. Naive lymphocytes recognize and respond to antigens during their circulation, and abnormalities in their migration and activation processes may be closely related to the genetic association between IBD and AS [76]. This suggests that the two diseases may share certain genetic bases that affect lymphocyte function, thereby exhibiting a degree of connection in their pathogenesis.

In conclusion, our team will pursue further investigations in the future, including in vitro and in vivo experiments, to validate the reliability of our findings. In addition, it is important to acknowledge certain limitations in our study, such as the lack of specified concentrations of the compounds used and the undetermined concentrations of the active ingredients in the final decoction composition. Drugs must be administered at specific concentrations to have their effects.

## 5. Conclusions

In summary, our research emphasizes that QJJP decoction may exert its therapeutic effects by influencing the relaxin signaling pathway, promoting osteoclast differentiation, modulating the HIF-1 signaling pathway, and interfering with the AGE-RAGE signaling pathway, with key target genes including IL1A, IFNG, TGFB1, and EDN1. This provides a solid theoretical basis for further experimental validation and clinical research. Particularly noteworthy is the role of IFNG in the HIF-1 signaling pathway, which will be a significant focus of our future investigations. Additionally, Mendelian randomization analysis further confirms a positive causal relationship between the occurrence of inflammatory bowel disease (IBD) and the development of ankylosing spondylitis (AS), enhancing the credibility of previous observational studies. Finally, we speculate that IFNG not only represents a key therapeutic target but also serves as a clinically meaningful diagnostic biomarker, potentially operating through genetic mechanisms.

## Figures and Tables

**Table 1:** Molecular docking of hub targets and key compounds.

Compound name	Target protein	PDB ID	Docking score (kcal/mol)
Quercetin	IL1A	5UC6	-6.94
	IFNG	1EKU	-7.406
	TGFB1	1KLA	-6.526

Oleic acid	EDN1	1EDN	-3.713

**Table 2:** MR estimates from each method of assessing the causal effects of IBD on AS.

Exposure	MR methods	Ankylosing spondylitis			
		Number of SNPs	OR (95% CI)	SE	MR *P* value
Inflammatory bowel disease	MR-Egger	57	1.00075 (1.00005~1.00145)	0.00036	0.0402
	Weighted median	57	1.00073 (1.00035~1.00112)	0.00020	0.0002
	Inverse variance weighted	57	1.00073 (1.00045~1.00101)	0.00014	4.11*E*-07
	Simple mode	57	1.00086 (1.00003~1.00169)	0.00042	0.0472
	Weighted mode	57	1.00103 (1.00039~1.00167)	0.00033	0.0027

**Table 3:** MR estimates from each method of assessing the causal effects of AS on IBD.

Exposure	MR methods	Inflammatory bowel disease	
		Number of SNPs	MR *P* value
Ankylosing spondylitis	MR-Egger	4	0.3480
	Weighted median	4	0.0589
	Inverse variance weighted	4	0.3780
	Simple mode	4	0.6655
	Weighted mode	4	0.2629

## Data Availability

The datasets utilized in this study are available in online repositories.

## Supplementary Materials

Supplementary Materials Table S1: detailed information of Mol ID and molecule name associated with herb name.
