# Supplementary material for: The Mechanism of “Treating Different Diseases with the Same Treatment” by Qiangji Jianpi Decoction in Ankylosing Spondylitis Combined with Inflammatory Bowel Disease: A Comprehensive Analysis of Multiple Methods
Source: Gastroenterol Res Pract. 2024 May 21;2024:9709260. doi: 10.1155/2024/9709260 (PMC11132832; doi:10.1155/2024/9709260)
Supplement: Supplementary Materials — Table S1: detailed information of Mol ID and molecule name associated with herb name. [file 9709260.f1.docx]

**Table S1：**Detailed information of Mol ID and Molecule Name associated with Herb name.

| Herb name | | Mol ID | Molecule Name |
| --- | --- | --- | --- |
| baishao | MOL001930 | | benzoyl paeoniflorin |
|  | MOL000359 | | sitosterol |
|  | MOL000358 | | beta-sitosterol |
|  | MOL000422 | | kaempferol |
|  | MOL001919 | | (3S,5R,8R,9R,10S,14S)-3,17-dihydroxy-4,4,8,10,14-pentamethyl-2,3,5,6,7,9-hexahydro-1H-cyclopenta[a]phenanthrene-15,16-dione |
|  | MOL001921 | | Lactiflorin |
|  | MOL001924 | | paeoniflorin |
|  | MOL000492 | | (+)-catechin |
|  | MOL000211 | | Mairin |
|  | MOL001910 | | 11alpha,12alpha-epoxy-3beta-23-dihydroxy-30-norolean-20-en-28,12beta-olide |
|  | MOL001928 | | albiflorin_qt |
|  | MOL001925 | | paeoniflorin_qt |
|  | MOL001918 | | paeoniflorgenone |
| baizhu | MOL000020 | | 12-senecioyl-2E,8E,10E-atractylentriol |
|  | MOL000021 | | 14-acetyl-12-senecioyl-2E,8E,10E-atractylentriol |
|  | MOL000022 | | 14-acetyl-12-senecioyl-2E,8Z,10E-atractylentriol |
|  | MOL000028 | | [α-Amyrin](https://old.tcmsp-e.com/molecule.php?qn=28) |
|  | MOL000033 | | (3S,8S,9S,10R,13R,14S,17R)-10,13-dimethyl-17-[(2R,5S)-5-propan-2-yloctan-2-yl]-2,3,4,7,8,9,11,12,14,15,16,17-dodecahydro-1H-cyclopenta[a]phenanthren-3-ol |
|  | MOL000049 | | 3β-acetoxyatractylone |
|  | MOL000072 | | 8β-ethoxy atractylenolide Ⅲ |
| baizhi | MOL001494 | | Mandenol |
|  | MOL001939 | | Alloisoimperatorin |
|  | MOL001941 | | Ammidin |
|  | MOL001942 | | isoimperatorin |
|  | MOL001956 | | Cnidilin |
|  | MOL002883 | | Ethyl oleate (NF) |
|  | MOL005789 | | neobyakangelico l |
|  | MOL005792 | | {5-[2'(R)-Hydroxy-3'-methyl-3'-butenyl-oxy]furocoumarin} |
|  | MOL005800 | | Byakangelicol |
|  | MOL005802 | | propyleneglycol monoleate |
|  | MOL005806 | | 4-[(2S)-2,3-dihydroxy-3-methylbutoxy]furo[3,2-g]chromen-7-one |
|  | MOL005807 | | sen-byakangelicol |
|  | MOL000358 | | beta-sitosterol |
|  | MOL000449 | | Stigmasterol |
|  | MOL000953 | | CLR |
|  | MOL001506 | | Supraene |
|  | MOL001749 | | ZINC03860434 |
|  | MOL002644 | | Phellopterin |
|  | MOL003588 | | Prangenidin |
|  | MOL003791 | | Linolein, 2-mono- |
|  | MOL007514 | | methyl icosa-11,14-dienoate |
|  | MOL013430 | | Prangenin |
| chuanniuxi | MOL012286 | | Betavulgarin |
|  | MOL012298 | | Rubrosterone |
|  | MOL000358 | | beta-sitosterol |
|  | MOL000098 | | quercetin |
| danggui | MOL000358 | | beta-sitosterol |
|  | MOL000449 | | Stigmasterol |
| dijingcao | MOL001002 | | ellagic acid |
|  | MOL000359 | | sitosterol |
|  | MOL000422 | | kaempferol |
|  | MOL006319 | | (2aR,3R,5aS,5bS,7aS,9S,11aR,12aS)-3-((S)-2,5-dimethyl-4-methylenehexyl)-2a,5a,8,8-tetramethylhexadecahydrocyclopenta[a]cyclopropa[e]phenanthren-9-ol |
|  | MOL006321 | | (3S,4S,5R,10S,13R,14R,17R)-4,10,13,14-tetramethyl-17-((R)-6-methyl-5-methyleneheptan-2-yl)-2,3,4,5,6,10,12,13,14,15,16,17-dodecahydro-1H-cyclopenta[a]phenanthren-3-ol |
|  | MOL006326 | | Ensaculin |
|  | MOL006327 | | sterculin A |
|  | MOL006331 | | 4',5-Dihydroxyflavone |
|  | MOL006332 | | (3S,4S,5R,10S,13R,14R,17R)-3-hydroxy-4,10,13,14-tetramethyl-17-((R)-6-methyl-5-methyleneheptan-2-yl)-3,4,5,6,12,13,14,15,16,17-decahydro-1H-cyclopenta[a]phenanthrene-7,11(2H,10H)-dione |
|  | MOL006334 | | (1R,3aR,5aR,5bR,7aS,9S,11aR,11bR,13aS,13bS)-1-(3-hydroxyprop-1-en-2-yl)-3a,5a,5b,8,8,11a-hexamethylicosahydro-1H-cyclopenta[a]chrysen-9-ol |
|  | MOL006337 | | (3S,7R,8S,9S,10R,13R,14R,17R)-17-((2R,5R)-5-ethyl-6-methylheptan-2-yl)-10,13-dimethyl-2,3,4,7,8,9,10,11,12,13,14,15,16,17-tetradecahydro-1H-cyclopenta[a]phenanthrene-3,7-diol |
|  | MOL006338 | | (2aR,3R,5aS,5bS,7aS,9S,11aR,12aS)-3-((S,Z)-6-ethoxy-2,6-dimethylhept-4-en-1-yl)-2a,5a,8,8-tetramethylhexadecahydrocyclopenta[a]cyclopropa[e]phenanthren-9-ol |
|  | MOL000098 | | quercetin |
| duhuo | MOL001941 | | [Ammidin](https://old.tcmsp-e.com/molecule.php?qn=1941) |
|  | MOL001942 | | [isoimperatorin](https://old.tcmsp-e.com/molecule.php?qn=1942) |
|  | MOL000358 | | [beta-sitosterol](https://old.tcmsp-e.com/molecule.php?qn=358) |
|  | MOL003608 | | [O-Acetylcolumbianetin](https://old.tcmsp-e.com/molecule.php?qn=3608) |
|  | MOL004777 | | [Angelol D](https://old.tcmsp-e.com/molecule.php?qn=4777) |
|  | MOL004778 | | [[(1R,2R)-2,3-dihydroxy-1-(7-methoxy-2-oxochromen-6-yl)-3-methylbutyl] (Z)-2-methylbut-2-enoate](https://old.tcmsp-e.com/molecule.php?qn=4778) |
|  | MOL004780 | | [Angelicone](https://old.tcmsp-e.com/molecule.php?qn=4780) |
|  | MOL004782 | | [[(1R,2R)-2,3-dihydroxy-1-(7-methoxy-2-oxochromen-6-yl)-3-methylbutyl] 3-methylbutanoate](https://old.tcmsp-e.com/molecule.php?qn=4782) |
|  | MOL004792 | | [nodakenin](https://old.tcmsp-e.com/molecule.php?qn=4792) |
| fangfeng | MOL000011 | | (2R,3R)-3-(4-hydroxy-3-methoxy-phenyl)-5-methoxy-2-methylol-2,3-dihydropyrano[5,6-h][1,4]benzodioxin-9-one |
|  | MOL011730 | | 11-hydroxy-sec-o-beta-d-glucosylhamaudol_qt |
|  | MOL011732 | | anomalin |
|  | MOL011737 | | divaricatacid |
|  | MOL011740 | | divaricatol |
|  | MOL001941 | | Ammidin |
|  | MOL011747 | | ledebouriellol |
|  | MOL011749 | | phelloptorin |
|  | MOL011753 | | 5-O-Methylvisamminol |
|  | MOL002644 | | Phellopterin |
|  | MOL000359 | | sitosterol |
|  | MOL000173 | | wogonin |
|  | MOL000358 | | beta-sitosterol |
|  | MOL001494 | | Mandenol |
|  | MOL001942 | | isoimperatorin |
|  | MOL003588 | | Prangenidin |
|  | MOL007514 | | methyl icosa-11,14-dienoate |
|  | MOL013077 | | Decursin |
| juhe | MOL000131 | | EIC |
|  | MOL000675 | | oleic acid |
| ganjiang | MOL002464 | | 1-Monolinolein |
|  | MOL002501 | | [(1S)-3-[(E)-but-2-enyl]-2-methyl-4-oxo-1-cyclopent-2-enyl] (1R,3R)-3-[(E)-3-methoxy-2-methyl-3-oxoprop-1-enyl]-2,2-dimethylcyclopropane-1-carboxylate |
|  | MOL002514 | | Sexangularetin |
|  | MOL000358 | | beta-sitosterol |
|  | MOL000359 | | sitosterol |
| sangjisheng | MOL000359 | | sitosterol |
|  | MOL000098 | | quercetin |
| weilingxian | MOL001663 | | (4aS,6aR,6aS,6bR,8aR,10R,12aR,14bS)-10-hydroxy-2,2,6a,6b,9,9,12a-heptamethyl-1,3,4,5,6,6a,7,8,8a,10,11,12,13,14b-tetradecahydropicene-4a-carboxylic acid |
|  | MOL002372 | | (6Z,10E,14E,18E)-2,6,10,15,19,23-hexamethyltetracosa-2,6,10,14,18,22-hexaene |
|  | MOL000358 | | beta-sitosterol |
|  | MOL000449 | | Stigmasterol |
|  | MOL005594 | | ClematosideA'_qt |
|  | MOL005598 | | Embinin |
|  | MOL005603 | | Heptyl phthalate |
| gancao | MOL001484 | | Inermine |
|  | MOL001792 | | DFV |
|  | MOL000211 | | Mairin |
|  | MOL002311 | | Glycyrol |
|  | MOL000239 | | Jaranol |
|  | MOL002565 | | Medicarpin |
|  | MOL000354 | | isorhamnetin |
|  | MOL000359 | | sitosterol |
|  | MOL003656 | | Lupiwighteone |
|  | MOL003896 | | 7-Methoxy-2-methyl isoflavone |
|  | MOL000392 | | formononetin |
|  | MOL000417 | | Calycosin |
|  | MOL000422 | | kaempferol |
|  | MOL004328 | | naringenin |
|  | MOL004805 | | (2S)-2-[4-hydroxy-3-(3-methylbut-2-enyl)phenyl]-8,8-dimethyl-2,3-dihydropyrano[2,3-f]chromen-4-one |
|  | MOL004806 | | euchrenone |
|  | MOL004808 | | glyasperin B |
|  | MOL004810 | | glyasperin F |
|  | MOL004811 | | Glyasperin C |
|  | MOL004814 | | Isotrifoliol |
|  | MOL004815 | | (E)-1-(2,4-dihydroxyphenyl)-3-(2,2-dimethylchromen-6-yl)prop-2-en-1-one |
|  | MOL004820 | | kanzonols W |
|  | MOL004824 | | (2S)-6-(2,4-dihydroxyphenyl)-2-(2-hydroxypropan-2-yl)-4-methoxy-2,3-dihydrofuro[3,2-g]chromen-7-one |
|  | MOL004827 | | Semilicoisoflavone B |
|  | MOL004828 | | Glepidotin A |
|  | MOL004829 | | Glepidotin B |
|  | MOL004833 | | Phaseolinisoflavan |
|  | MOL004835 | | Glypallichalcone |
|  | MOL004838 | | 8-(6-hydroxy-2-benzofuranyl)-2,2-dimethyl-5-chromenol |
|  | MOL004841 | | Licochalcone B |
|  | MOL004848 | | licochalcone G |
|  | MOL004849 | | 3-(2,4-dihydroxyphenyl)-8-(1,1-dimethylprop-2-enyl)-7-hydroxy-5-methoxy-coumarin |
|  | MOL004855 | | Licoricone |
|  | MOL004856 | | Gancaonin A |
|  | MOL004857 | | Gancaonin B |
|  | MOL004860 | | licorice glycoside E |
|  | MOL004863 | | 3-(3,4-dihydroxyphenyl)-5,7-dihydroxy-8-(3-methylbut-2-enyl)chromone |
|  | MOL004864 | | 5,7-dihydroxy-3-(4-methoxyphenyl)-8-(3-methylbut-2-enyl)chromone |
|  | MOL004866 | | 2-(3,4-dihydroxyphenyl)-5,7-dihydroxy-6-(3-methylbut-2-enyl)chromone |
|  | MOL004879 | | Glycyrin |
|  | MOL004882 | | Licocoumarone |
|  | MOL004883 | | Licoisoflavone |
|  | MOL004884 | | Licoisoflavone B |
|  | MOL004885 | | licoisoflavanone |
|  | MOL004891 | | shinpterocarpin |
|  | MOL004898 | | (E)-3-[3,4-dihydroxy-5-(3-methylbut-2-enyl)phenyl]-1-(2,4-dihydroxyphenyl)prop-2-en-1-one |
|  | MOL004903 | | liquiritin |
|  | MOL004904 | | licopyranocoumarin |
|  | MOL004905 | | 3,22-Dihydroxy-11-oxo-delta(12)-oleanene-27-alpha-methoxycarbonyl-29-oic acid |
|  | MOL004907 | | Glyzaglabrin |
|  | MOL004908 | | Glabridin |
|  | MOL004910 | | Glabranin |
|  | MOL004911 | | Glabrene |
|  | MOL004912 | | Glabrone |
|  | MOL004913 | | 1,3-dihydroxy-9-methoxy-6-benzofurano[3,2-c]chromenone |
|  | MOL004914 | | 1,3-dihydroxy-8,9-dimethoxy-6-benzofurano[3,2-c]chromenone |
|  | MOL004915 | | Eurycarpin A |
|  | MOL004917 | | glycyroside |
|  | MOL004924 | | (-)-Medicocarpin |
|  | MOL004935 | | Sigmoidin-B |
|  | MOL004941 | | (2R)-7-hydroxy-2-(4-hydroxyphenyl)chroman-4-one |
|  | MOL004945 | | (2S)-7-hydroxy-2-(4-hydroxyphenyl)-8-(3-methylbut-2-enyl)chroman-4-one |
|  | MOL004948 | | Isoglycyrol |
|  | MOL004949 | | Isolicoflavonol |
|  | MOL004957 | | HMO |
|  | MOL004959 | | 1-Methoxyphaseollidin |
|  | MOL004961 | | Quercetin der |
|  | MOL004966 | | 3'-Hydroxy-4'-O-Methylglabridin |
|  | MOL000497 | | licochalcone a |
|  | MOL004974 | | 3'-Methoxyglabridin |
|  | MOL004978 | | 2-[(3R)-8,8-dimethyl-3,4-dihydro-2H-pyrano[6,5-f]chromen-3-yl]-5-methoxyphenol |
|  | MOL004980 | | Inflacoumarin A |
|  | MOL004985 | | icos-5-enoic acid |
|  | MOL004988 | | Kanzonol F |
|  | MOL004989 | | 6-prenylated eriodictyol |
|  | MOL004990 | | 7,2',4'-trihydroxy－5-methoxy-3－arylcoumarin |
|  | MOL004991 | | 7-Acetoxy-2-methylisoflavone |
|  | MOL004993 | | 8-prenylated eriodictyol |
|  | MOL004996 | | gadelaidic acid |
|  | MOL000500 | | Vestitol |
|  | MOL005000 | | Gancaonin G |
|  | MOL005001 | | Gancaonin H |
|  | MOL005003 | | Licoagrocarpin |
|  | MOL005007 | | Glyasperins M |
|  | MOL005008 | | Glycyrrhiza flavonol A |
|  | MOL005012 | | Licoagroisoflavone |
|  | MOL005013 | | 18α-hydroxyglycyrrhetic acid |
|  | MOL005016 | | Odoratin |
|  | MOL005017 | | Phaseol |
|  | MOL005018 | | Xambioona |
|  | MOL005020 | | dehydroglyasperins C |
|  | MOL000098 | | quercetin |
